# The Relation of Membranous Croup to Diphtheria

**Published:** 1880-03

**Authors:** J. O. Roe

**Affiliations:** Rochester, N. Y.


### THE BUFFALO

### MEDICAL AND SURGICAL JOURNAL.

Vol. XIX.—MARCH, 1880.—No. 8.

### Original Communications.

### THE RELATION OF MEMBRANOUS CROUP TO DIPHTHERIA.

BY J. O. ROE, M. D., ROCHESTER, N. Y.

There is no subject in modern medicine which has recently received a greater amount of attention, and concerning which wider opinions are held, than the relation of membranous croup to diphtheria.

Some can only discern one common disease with a variety of manifestations; while others see in each an independent and distinct affection.

Diphtheria, as a disease, has evidently existed since the earliest times. In the examination of the oldest writings of medical authors we find it clearly described, although under nearly as many different names as there were authors alluding to it. Diphtheria is supposed to be the disease alluded to in passages of Homer, and it is quite clearly traced to the time of Pythagoras (580-489 B. C.) Hippocrates mentions it in his writings, but to Bennett, in the early part of the 7th century, is due the honor of first describing it as it exists at the present time. The

* A paper read before the Monroe Co. Medical Society.

syriac ulcer or the malum aegyptaicum as described by Aretaeus, the askara frequently mentioned in the Talmud; later the morbus strangulatorium, male in canna, and the morbi suffocantis garrotillo which prevailed in Spain between the years 1581 and 1611, were undoubtedly the same disease; as all are described as highly contagious affections of the throat of a very fatal character.

By Aretaeus it was also described as a cynanchia (or dog-choke,) and existed in two forms, an inflammation of the respiratory organs, and an affection of the breath. In the first form it affected the tonsils, epiglottis, pharynx, uvula, and top of wind-pipe. In the second, the organs became collapsed and a sense of stifling, so violent, that it seemed to the patient as if the inflammation was secretly concealed in his chest, in the vicinity of the heart and lungs. This second form he termed "internal choke" and considered it merely a disease of the breath. (Ætiology and Semiology, Renold's translation, p. 13).

The above is in substance the opinion held in regard to the disease, and the term "cynanche maligna" was universally applied to it, until a century ago, when in 1765 an epidemic of the disease appeared on the eastern coast of Scotland, in which the laryngo-tracheal symptoms bore so conspicuous a part and differed so much from those affecting the pharynx alone, that it was described by Dr. Home under the name of "croup"—a Scotch word, first introduced into medical literature by Dr. Blair of Cupar Angus, in 1713.

This new disease, as it was then called, attracted considerable attention from the profession, and, assisted by the able memoir of Dr. Home, produced so deep and lasting an impression, that many of our most able writers and accurate observers have, with great reluctance, been compelled to adopt the original and evidently correct idea of the unity of the two affections.

The word "croup" or "croups," was used by the populace previous to the time of Home; but chiefly applied, as at the present day, to cases of "laryngitis stridulous," now known as false or spurious croup.

The two diseases were considered as independent affections until the time of Bretinneau, who was the first to give the name diphtheria to a membranous formation found in the throats of those who were attacked by the disease during the outbreak at Tours, in 1818.

The appearance of the common characteristic, a membranous formation, led many to believe in a common origin for the two diseases: the teachings of Bretinneau, supported by his distinguished pupil Trousseau, so influenced the leading French pathologists that the unity of the two diseases has since been pretty generally taught.

The points of difference on which the advocates of the duality theory base their conclusions are:

1. The supposed pathological difference.
2. The alleged clinical difference.

In 1847, Virchow, afterwards Wagner, Rockitansky, Buhl and Riendfleisch, attempted to find the distinction between the membrane of the two diseases in the appearance and anatomical constituents of the exudate. They and their followers were soon compelled to abandon all such attempts, for in the examination of membranes from the two, as they supposed, unquestionably different sources they were unable to find any chemical, microscopical, or general distinction to indicate their origin. Not being willing to relinquish altogether their views as to a pathological difference, they then sought to explain it by the manner in which the exudate was thrown out. In croup it was poured out in a liquid form and coagulated on the surface of the membrane, and could be readily removed, leaving, perhaps, a hyperaemic but smooth surface; while in diphtheria the mucous membrane was incorporated more or less with the exudate, that it was poured into the substance of the tissue, and on removal left a raw and bleeding surface.

This supposed distinction had soon to be abandoned under the light of investigation, for it was amply explained by the difference in the structure of the parts, and the length of time which the exudate had been thrown out.

In the earlier stages of a membranous formation the exudate is easily pealed off and removed; but in the latter stages there is more or less destruction of the superficial tissues and muciparous glands, so that the mucous, which is the active agent of lifting the membrane, is not secreted; and the exudate penetrates more deeply into the tissues. Now, in croup, it is during this early stage that the larynx and trachea become blocked, and suffocation takes place before the second stage is reached.

Having thus failed to establish an anatomical or pathological distinction, it was then sought for in a clinical difference based on the following phenomena. The universal and characteristic symptom of croup has been considered to be the presence of a false membrane, in contra-distinction to diphtheria, in which it may be absent, and to false croup which includes laryngitis stridulous.

These were the views of the French pathologists, except that it was considered a form of diphtheria. The failure to find a false membrane in patients who had died of typical croup began to shake the belief of many and caused them to seek for the explanation of this apparent paradox. Rayland and Sir Thomas Watson mention the occurrence, and Geo. Johnson was much surprised to find on a post-mortem examination of two unquestionable cases of croup, no appearance of a false membrane in any part of the air passages. (*Lancet*, April 26, 1879, p. 592.) This phenomenon is explained by Steiner, by the possibility of a membrane being present during life, in a fluid or coagulated form, but expectorated before death. (Ziemsson's Cyclopædia, vol. iv., p. 256.)

Again it is considered that diphtheria is a disease of the pharynx and spreads upward to the nares, and only occasionally extends to the larynx and trachea, while croup is regarded as a disease primarily affecting the larynx, and is confined to this region and to the trachea. "The fact is," says Mackenzie, "that croup is a disease which commonly commences in the pharynx, and only in about 10 or 12 per cent. of cases originates in the larynx and trachea." (Diphtheria: Its Nature and Treatment, London, 1879, p. 83.)

The third and strongest point on which the duelists fix their faith is the theory, that croup is local and is not contagious, while diphtheria is a general or constitutional disease and is contagious.

A glance at the anatomy of this region will readily explain the limited amount of constitutional disturbance, on the ground that the general symptoms are secondary and commensurate with the local processes.

In all systemic diseases attended by local manifestation, the extent of the local symptoms is, pari passu, proportionate to the general disturbance, and if the disease be light the local evidence is often wanting altogether. This is true of scarlet fever, measles, and sometimes even of small-pox.

The parts most readily and violently attacked by the diphtheritic imflammation are those covered with pavement epithelium, and most scantily supplied with muciparous follicles. This is owing to the fact that pavement epithelium is lower in the scale of animal life, that vibrating epithelium will resist destructive changes longer, and when there is an abundant supply of muciparous glands the pouring out freely of the normal secretion prevents as a rule deep-seated degeneration of tissue.

When the local diphtheritic inflammation attacks the tonsils or pharynx as it usually does, it extends by continuity of structure over the surface covered by the pavement epithelium; but at the entrance of the larynx it is met by the sentinels, the little resisting cilia of the epithelium covering the ventricular bands or false vocal cords and the venticles.

If the disease has not sufficient force, they are equal to the emergency of arresting its progress in this direction. If the inflammation attempts to travel upward, it is met by the same opposing force in the lower portion of the nasal cavity. If, however, the disease is raging with sufficient energy to overcome these barriers, a general nasal laryngeal and tracheal diphtheria is the result, always of a very grave character, not only from its extensive local implication, but as indicative of the degree of systemic affection.

When the local manifestation first appears in the pavement epithelium covering the vocal cords, it is naturally confined to a very limited area. If it attempt to surmount into the pharynx it is resisted by the little cilia of the ventricular bands. If it attempt to extend itself into the bronchia, it is resisted by the cilia there, though less strongly owing to the thinness of the membrane. So in the distribution of the tissue which has the least resisting power to the destructive poison, we find the solution of its usual limitation to the pharynx and tonsils, when it chooses the larynx for its first invasion, it obstructs the gate-way of life so quickly that in many cases death takes place before the attendant train of symptoms, as asthenia, implication of the lymphatics, albuminuria, etc., have had time to appear.

The fact of the greater liability of the implication of the lymphatics of the neck in pharyngeal and nasal diphtheria, than in croup or tracheal diphtheria, is owing to the distribution of the lymphatic communications.

The tonsils have no connection with the lymphatic system, but the tongue, uvula, soft palate, anterior and posterior pillars of the pharynx, cheeks, lips and the lower portion of the nasal cavity, contain numerous lymphatics, which connect with the deep facial, deep cervical, submaxillary and finally the supra-clavicular and jugular plexus. In the larynx and trachea are found no lymphatic glands and but few lymphatic vessels. These latter terminate in the solitary glands at the side of the trachea, and do not communicate with the general lymphatic system. The trachea is also abundantly supplied with muciparous follicles, which pour out their secretion, lift the exudate, and cause the easy separation of the tracheal membrane without affecting the surface beneath.

The rarity of albuminuria in croup is, as already mentioned, due to the slight amount of lymphatic implication and to the extremely fatal character of the disease; death usually occurring from suffocation before there is sufficient time for albuminuria to become established, and if present in these cases, it is not often looked for.

The great fatality in children is due to the smallness of the larynx, the greater number and size of the lymph vessels, the frequency of catarrh of the nares and also of the mouth from lack of cleanliness and enlarged tonsils.

In a family, which I attended recently, a child died of laryngeal diphtheria, with no pharyngeal manifestations. The father and mother were taken shortly after with membranous laryngitis, but recovered. This case would ordinarily have been considered a genuine case of croup.

It is claimed for croup, that it is a non-contagious malady, while dyphtheria is a markedly contagious disease. The belief in the non-contagious character of so-called membranous croup, is based on the frequent occurrence of cases, where there was no apparent exposure to any diphtheritic infection, and where one member of a family of children is attacked without its being communicated to the others.

To show that diphtheria is a contagious disease, no argument or proof is required, but to say that exceptions are not constantly occurring, would be to deny what is almost a daily observation.

When diphtheria has died out as an epidemic, the stray cases with limited infecting power will be known for years or decennia as so-called sporadic membranous croup, as one would speak for a generation of an occasional case of spasmodic cholera, or a stray case of variola. There is not infection enough to poison the throat, and larynx and blood, but just sufficient for the most favorable place, the vocal cords.

Mackenzie says: "The dangers which are most to be dreaded at the outset of an attack are on the one hand, extension of the disease to the larynx, and on the other, severe blood poisoning." Again he says, "diphtheria is, for obvious reasons, far more fatal amongst children than adults," and again, "when the exudation shows a disposition to extend rapidly, the danger is very considerable as the extension is most likely to take place in the direction of the larynx." (Diphtheria: Its Nature and Treatment, p. 64.)

To cite more evidence to show that the chief symptom in the earliest stages, of a serious import, is the tendency to the involvement of the larynx, is to reiterate what is our daily observation during epidemics of diphtheria; and when it becomes thus early involved, the symptoms are so nearly identical with those produced by so-called "true membranous croup" that the attempt to distinguish between the two, says Johnson, is hopeless and most confusing to the student, for it is certain that membranous croup and laryngeal diphtheria, as we now see them, are one and the same malady. (*London Lancet,* 1875, vol. 1, p. 81.)

The cause of the more frequent recoveries from tracheotomy in croup than in diphtheria is due to the fact, as has already been shown, that the cause of death in croup is from obstruction of the larynx and trachea by the membrane.

It is claimed for croup that it is a sthenic disease, while diphtheria is asthenic and attended by more systemic depression. A study of the cases which come under our observation, and a glance at the disease as described in works of practical medicine, will at once convince us of the error of this assumption.

In order to settle the question as to the relationship of the two diseases, last winter the Royal Medical and Chirurgical Society appointed a committee to conduct the investigation. To obtain sufficient data on which to base a conclusion, a circular was issued with a complete list of questions concerning all the points at issue. To these ninety replies were received mostly from men of extended observation and experience; besides all the records of hospitals, and information from every possible source was obtained. The following is a synopsis of the conclusions which were based on the evidence furnished.

1st. Membranous inflammation confined chiefly to or affecting the larynx and trachea may arise.

*a.* From the diphtheritic contagion.

*b.* By means of foul water, air, or other agents.

*c.* As accompanying measles, scarlet fever, typhoid fever, etc.

*d.* From various accidental causes of irritation, as the inhalation of hot water, steam, contact of acids, etc.

2d. Following or associated with exposure to cold, but not to exclude the possible co-existence of other causes.

3d. Membranous inflammation chiefly of the larynx and trachea to which the term membranous croup would commonly be applied, may be imparted by an influence, epidemic or of other sort, which in other persons has produced pharyngeal diphtheria.

4th. And conversely, a person suffering with the membranous affections chiefly of the air passages, such as would commonly be termed membranous croup, may communicate to another a membranous condition limited to the pharynx and tonsils, which would be regarded commonly as diphtheria. From these facts the committee conclude that membranes, chiefly laryngeal, are so often associated with similar ones on the fauces, and such general constitutional condition that no line of demarcation can be drawn, except that when the pharynx is primarily affected, constitutional disturbance is more marked.

All the points at issue have been gradually relinquished by those who have advocated the non-identity of the two affections, until the dispute is narrowed down to the question whether "Membranous laryngitis is always caused by the special poison of diphtheria."

In regard to this question what can be more explicit, when in section 3 and 4 the committee says, that membranous croup may be imparted by an influence, which in other persons has produced pharyngeal diphtheria, and conversely a person suffering from "membraneous croup" may communicate to another diphtheria.

In order to reconcile those whose views differed so widely on this subject, and to form a more uniform basis for reports in the future, in conclusion, the committee suggests that the term croup be *henceforth used* wholly as a clinical definition, implying laryngeal obstruction occurring with febrile symptoms in children. Thus croup may be membranous or not membranous, due to diphtheria or not so, and diphtheria may or may not be attended with croup. The committee, therefore, propose that the term membranous laryngitis should be employed in order to avoid confusion, whenever the knowledge of the case is such as to allow of its application. (Report of Committee, p. 31—33, 1879.)

This seems an eminently wise conclusion, and by it the original Scotch word, croups or croup, is restored to its primitive signification, as describing the purely subjective symptoms of laryngismus stridulus, laryngeal spasm or spurious croup, so common to children, and not the objective condition of a membranous formation.

It would be interesting to follow through the course of this debate, which took place in the Royal Medical and Chirurgical Society, on this most important question, but space will not permit. There is one question about which many are in doubt, it is the relation of membranous formation in the fauces after exposure to colds, foreign substances, and chemical agents, and the occurrence of false membranes on wounds, burns and denuded surfaces. On a close examination of these cases it was found that these accidental occurrences were associated with a special condition of the system. If it was the rule for wounds and abraded surfaces to become covered with a membrane, then it could not be explained by a diphtheritic origin, but as the occurrence is a rare exception, it is no more unreasonable to attribute it to a diphtheritic (if we might so call it) condition of the system or diphthertic complication than to ascribe certain symptoms supervening with another disease to typhoid complication.

In consulting the older works on this subject one would be led to consider membranous croup a very frequent and fatal malady. Tanner (*Prac. Med.*, p. 501) estimates that one in twelve children died of this disease. He found in London, in 1866, the fatal cases attributed to this disease amounted to 5,168, of which 2,706 were males and 2,462 females; while the committee has, from all the reports of hospitals, dispensaries and private records, been able to collect only 49 cases, which could be properly considered as primary membranous croup. This very great and increasing rarity of this disease is undoubtedly due, as suggested by Dr. Poore (*Lancet*, May 3, 1879, p. 630,) to increasing knowledge of diphtheria.

The last, and perhaps the most plausible theory, if it could be shown to be true, is the bacterian or germ theory for the local cause of diphtheria as a distinction from membranous croup.

By Brettineau. the active contagious principle was considered to reside in the secretions from the membranous exudate alone; that without an exudation there could be no diphtheria; that it only spread in the form of dust like atoms and could only be conveyed by inoculation.

These views were not universally accepted, for most physicians recognized its infectious character and believed, as with the majority of physicians at the present day, it to be a systemic disease.

Laycock, in 1858, and Jodin in 1859, were the first to demonstrate the presence of parasitic elements in the diphtheritic slough or false membrane.

Buhl, in 1867, also drew attention to the same fact, and shortly after Oertel and Heuter discovered that the membrane and secretions of the subjacent diseased parts, and even of the blood of persons sick with diphtheria, contained in great numbers vegetable organisms or bacteria, to which Oertel gave the name of micrococci.

In accordance with this belief in the causative agent of the disease, Oertel declares that diphtheria begins as a local disease and develops often into a general one, and that, moreover, the general infection is kept up by the local one. The disease establishes itself at first in one spot, the focus of infection, and from thence radiates, as it were, through the body, until by general blood poisoning it renders the organism incapable of life. (Ziemssen Cyclopædia, vol. 1, p. 581.)

Thus, the bacterian theory of disease is invoked as a distinction to differentiate the two affections, for it is contended that in croupous membranes bacteria are never found, but if so, they are purely accidental and play no part as a causative element in the disease.

The presence of bacteria in the pharyngeal diphtheritic exudate is proven beyond question; but it is nevertheless true that they are of very frequent occurrence in the laryngeal or tracheal exudate of croup, and they are also found swarming in the mucous of these membranes, when in an undoubted state of health.

According to the bacterian theory, diphtheria is not a general blood-poisoning or a previously existing disease, which first produces the membrane, but it is these parasites which are drawn in with the inspired air and lodge on the surface, which accounts for its usually choosing the throat as the seat of the primary local manifestation; indeed, Eberth goes so far as to declare that "without micrococci there can be no diphtheria." (Zur Kenntn. der Bacterit. Mycosen, Leipz., 1872.)

That diphtheria is a filth disease but few will deny, as nearly all cases are found, when occurring sporadically, to be associated with some local surroundings, in which hygiene is disregarded or, when occurring as an epidemic, to some general atmospheric poisoning. The analogy, which diphtheria bears to typhoid fever in its mode of development and propagation, is very striking. In typhoid fever the disease has a special selection for Payers' Patches; while in diphtheria the throat and bronchial mucous membrane is the chosen site.

We all recognize the relation of acute infectious diseases to heat, cold, moisture, and the impregnation of the atmosphere with the emanations from decaying animal and vegetable tissue, but to attribute the phenomena of diphtheria and all the specific fevers, as has been attempted, to the presence of germs, (Dr. Maclagan on Germ Theory, London, 1876,) the proof is not sufficiently positive to warrant such a conclusion.

If the presence of germs explain the origin of these diseases and particularly of diphtheria, then we should be able by means of the microscope to detect the cause of the mildness or virulence of cases, or epidemics of diphtheria to reside in the prevalence of these germs in greater or less number, or in a greater or less size and activity of the germs.

This has been attempted by many eminent microscopists, but all their efforts have thus far proven utterly futile.

In order to prove that in these germs reside the contagium vivum, Oertel attempted to prove by a series of experiments on lower animals, that diphtheria fixes itself at the point of inoculation and radiates from that place throughout the body.

This same fact is true of syphilis and vaccine virus, but who will attempt to say that syphilis is of bacterian origin, and with vaccine virus it is postively shown not to be the case.

In order to determine if possible the nature of the infectious principle of diphtheria and the circumstances that determine the infection, a series of experiments were instituted by the New York Board of Health, under the direction of Drs. Thos. E. Satterthwaite and Edward Curtis.

The first series of experiments were made by inoculating rabbits with diphtheritic membrane. Thirty-eight were inoculated, of which twenty-two died, but not with symptoms resembling diphtheria.

Inoculations were then made with scrapings from the upper surface of a somewhat furred tongue from a healthy person, and which swarmed with bacteria. The effect in these were similar to those from the diphtheritic membrane. Chohen's fluid, which had become putrid, was then used with similar effect but in a less degree. In order to exclude the possibility of a local cause inoculations were made with sand, but with negative results.

To determine, if possible, whether the poisonous principle resided in the fluid or solid elements, the infectious infusion was filtered through porous clay; with the filtrate, which was perfectly clear and inodorous, inoculations were made, but with entirely negative results, while the residue or the unfiltered infusion was found in all cases to be more or less virulent. It being determined that the infectious element resided in the residue or solid portion, the next step was to determine if it was especially connected with the bacteria formed in the infectious material. This was accomplished in two ways:

1st. By arresting the propagation of bacteria by salicylic acid.

2d. By destroying them altogether by a prolonged temperature of 212° Fahr. and boiling alcohol, and then placed under circumstances favorable to the development of bacteria.

Inoculations made with the infusions thus treated continued equally as poisonous as before, although in many instances no bacteria were developed or could be detected. Hence it was determined that "the granules, which are the bodies most likely to represent the poisonous principles, appear not to be bacteria or their spores." (Satterthwaite, *N. Y. Med. Record*, 1875, p. 853. Also report, New York, Feb. 11th, 1877.)

To confirm the above experiments, vaccine virus was mixed with salicylic acid, which prevented the appearance of bacteria and decomposition, and preserved the lymph in a fresh state for a long time, but did not lessen in the least its active effects. The same was also found by Dr. Curtis to result when carbolic acid was used.

After the discovery of torulae in fermenting substances by Pasteur, it was held by him and others that fermentation and putrefaction were always initiated by organisms, until Bastain (*London Lancet*, April 10, 1875, p. 508) showed that "grapes suspended in an atmosphere of carbonic acid, undergo fermentation so as to generate alcohol and other products, even without the presence of torula or other organisms."

Notwithstanding the above facts, there can be little doubt that these organisms which are so generally found associated with fermentive and decomposing processes, bear a certain relation to diseased processes. Not that they are capable of inducing disease *per se*, but that they are intimately associated with conditions which produce it ( as has been shown by Bastain, Panum Onimus, Hiller, Chauveau, Curtis, Satterthwaite and others,) and their presence in greater numbers and activity simply indicate the extent of the unfavorable conditions which give rise to their development.

Thus these facts adduced in opposition to the germ theory in general are equally available as objections to the bacterian origin of diphtheria as a distinction from membranous croup.

In the debate upon the germ theory of disease which occurred in the Pathological Society of London, in April and May, 1875, ( *London Lancet*,) it was strenuously opposed by Carlton Bastain, in his very able and scholarly address on opening the debate and in the discussion by Geo. Johnson, Lionel S. Beale and by Murchison and Dougall of Glasgow, and others.

Dr. Burdon Sanderson who had supported the germ theory since 1870, on what he then considered to be the strongest grounds in its favor, viz : " the theory that in these processes of disease in which minute organisms are found, the life which interferes in these processes is not only the life of the tissues themselves, which are the seat of disease, but another kind of life is introduced."

He said that, " notwithstanding the theoretical change which has taken place in regard to the inseparable connection, which bacteria seems to have to disease and the doubt which has arisen as to whether they were a cause or consequent of morbid processes, we should not put away facts, we should still keep to the investigation of facts, clinical facts and pathological facts as our principal object ; He also added, " supposing any one of us went to Ricklinghausen, Virchow, Chaveau, Paget, and ask the question, 'Do you believe in the germ theory?' the answer he would certainly get would be, 'I really cannot give you any opinion upon the subject. A great number of observations have been made upon the subject; you must read these observations; then, if you wish to pursue it you must make observations yourself, and perhaps, at a future time it may be possible to come to a conclusion upon this subject.' But if they were pressed to give an answer to the question, 'Do you believe in the germ theory?' I believe all these eminent men would shrug their shoulders."

The question naturally presents itself, if these minute germs of vegetable life are the causative agents in diphtheria, why should they limit their operations to the tonsils and parts adjacent, while all other portions of the respiratory tract escape in the great majority of instances? for it has not been demonstrated that the parts covered by pavement epithelium are a more fertile feeding ground for bacteria than other surfaces.

"Surely," says Lyons opt. cit. (proceedings of the Connecticut Medical Society, 1876) "these germs as they are swept along over the fourteen hundred to two thousand square feet of respiratory mucous membrane, ought to be able to obtain a foothold and to produce irritation and inflammation in more than one particular place, unless Oertel, Heuter and others have greatly overrated their clinging, boring and other pernicious properties. Finally, it appears impossible to explain cases of diphtheria terminating fatally in 24, 36 and 48 hours, with little or no membrane in the throat upon the bacterian theory, which would seem to require more time for its fatal operation than is thus allowed.

"It must, therefore, be acknowledged that this brilliant theory, though begotten within the temple of science, and fostered by her ablest experts, is yet quite unable to stand the tests of experimental investigations, or of too feeble growth to withstand adverse criticism, or to cope with its older rival, the chemical theory."

